# Lean body mass: the development and validation of prediction equations in healthy adults

**DOI:** 10.1186/2050-6511-14-53

**Published:** 2013-10-14

**Authors:** Solomon Yu, Thavarajah Visvanathan, John Field, Leigh C Ward, Ian Chapman, Robert Adams, Gary Wittert, Renuka Visvanathan

**Affiliations:** 1 Aged and Extended Care Services, Level 8B Main Building, The Queen Elizabeth Hospital, Central Adelaide Local Health Network, 21 Woodville Road, 5011 Woodville South, SA, Australia; 2 Adelaide Geriatrics Training and Research with Aged Care (G-TRAC) Center, School of Medicine, University of Adelaide, South Australia, Australia; 3 Discipline of Medicine, School of Medicine, Faculty of Health Science, University of Adelaide, Adelaide, SA, Australia; 4 Department of Anaesthesia, The Queen Elizabeth Hospital, Central Adelaide Local Health Network, South Australia, Australia; 5 School of Chemistry and Molecular Biosciences, The University of Queensland, Brisbane, QLD, Australia; 6 The Health Observatory, Discipline of Medicine, School of Medicine, Faculty of Health Science, University of Adelaide, Adelaide, South Australia, Australia

**Keywords:** Lean body mass, Weight, Older people, Drugs

## Abstract

**Background:**

There is a loss of lean body mass (LBM) with increasing age. A low LBM has been associated with increased adverse effects from prescribed medications such as chemotherapy. Accurate assessment of LBM may allow for more accurate drug prescribing. The aims of this study were to develop new prediction equations (PEs) for LBM with anthropometric and biochemical variables from a development cohort and then validate the best performing PEs in validation cohorts.

**Methods:**

PEs were developed in a cohort of 188 healthy subjects and then validated in a convenience cohort of 52 healthy subjects. The best performing anthropometric PE was then compared to published anthropometric PEs in an older (age ≥ 50 years) cohort of 2287 people. Best subset regression analysis was used to derive PEs. Correlation, Bland-Altman and Sheiner & Beal methods were used to validate and compare the PEs against dual X-ray absorptiometry (DXA)-derived LBM.

**Results:**

The PE which included biochemistry variables performed only marginally better than the anthropometric PE. The anthropometric PE on average over-estimated LBM by 0.74 kg in the combined cohort. Across gender (male vs. female), body mass index (< 22, 22- < 27, 27- < 30 and ≥30 kg/m^2^) and age groups (50–64, 65–79 and ≥80 years), the maximum mean over-estimation of the anthropometric PE was 1.36 kg.

**Conclusions:**

A new anthropometric PE has been developed that offers an alternative for clinicians when access to DXA is limited. Further research is required to determine the clinical utility and if it will improve the safety of medication use.

## Background

With increasing age, there is a decline in lean body mass (LBM) and very often an increase in adiposity [1]. The decline in LBM may also be accompanied by a reduction in physical function and when a pathological threshold is reached, the person is said to have sarcopenia [2]. In recent times, sarcopenia has been recognized as an independent predictor of drug related adverse outcomes in the oncology setting where muscle wasting can be common [3,4]. Drug-related adverse effects are defined as medical events related to the use of medication which may result in disability, hospital admissions or death [5]. In patients with cancer, the use of LBM might be superior to body surface area (BSA) [6]. For example, in a prospective study of colon cancer patients treated with 5-fluorouracil (5-FU), the incidence of dose limiting toxicity was examined with respect to conventional dosing of 5-FU/m^2^ of BSA versus 5-FU/kg of LBM. LBM was a better predictor of toxicity (p = 0.011) but not BSA [6]. Similar findings have been reported in other studies [7,8]. In anaesthesia, propofol pharmacokinetic parameters scaled linearly to LBM is also said to provide for improved dosing in adults [9]. Therefore, accurate measurement of LBM may have clinical application in improving drug prescribing safety and efficacy, especially in older people where loss of lean mass is common.

A major impediment to the routine clinical use of LBM is the reliance on relatively inaccessible or expensive methods of body composition measurements. Computed tomography (CT), magnetic resonance imaging and dual absorptiometry x-ray (DXA) are used to assess LBM but these methods may be difficult to access in clinical practice (e.g. frail or rural patients) [10]. Although the bioelectrical impedance analysis (BIA) method is portable, it still requires the purchase of special equipment and it’s accuracy is also dependent on many other factors such as state of hydration, food intake and exercise [11].

Total body weight consists of fat mass and fat free mass. Fat free mass (FFM) consists of bone, muscle, vital organs and extracellular fluid. LBM differs from FFM in that lipid in cellular membranes are included in LBM but this accounts for only a small fraction of total body weight (up to 3% in men and 5% in women) [12]. In the literature, bone mass has at times been included in LBM and at other times not included [4,13].

Anthropometric-based prediction equations (PEs) have been examined as an alternative in measuring LBM in settings where access to these accurate methods is limited. In a very recent study of older (≥70 years) Australian men, FFM as estimated by three PEs were compared to FFM as estimated by DXA (FFM_DXA_) [14]. The three PEs were the Heitmann, Janmahasatian and Deurenberg equations as shown below:

*Heitmann equation*[15]*:*

$$\begin{array}{c} \text{Body}\;\text{fat}\;{\left(\text{kg}\right)}_{\text{male}}=\left(0.988\;\times \;\text{BMI}\right)\\ \;+\left(0.242\;\times \;\text{weight}\right)\\ \;+\left(0.094\;\times \;\text{age}\right)-30.180 \end{array}$$

$$\begin{array}{c} \text{Body}\;\text{fat}\;{\left(\text{kg}\right)}_{\text{female}}=\left(0.988\;\times \;\text{BMI}\right)\\ \;+\left(0.344\;\times \;\text{weight}\right)\\ \;+\left(0.094\;\times \;\text{age}\right)-30.180. \end{array}$$

*Janmahasatian equation*[12]*:*

$$\begin{array}{c} \mathrm{FFM}\;{\left(\mathrm{kg}\right)}_{\mathrm{female}}=\left(9270\;\times \;\mathrm{weight}\right)\\ \;/(8780+\left(244\;\times \;\mathrm{BMI}\right) \end{array}$$

$$\begin{array}{c} \mathrm{FFM}\;{\left(\mathrm{kg}\right)}_{\mathrm{male}}=\left(9270\;\times \;\mathrm{weight}\right)\\ \;/(6680+\left(216\;\times \;\mathrm{BMI}\right) \end{array}$$

*Deurenberg equation*[16]*:*

$$\begin{array}{c} \mathrm{Body}\;\mathrm{fat}\left(\%\right)=\left(1.2\;\times \;\mathrm{BMI}\right)\\ \;+\left(0.23\;\times \;\mathrm{Age}\right)–\left(10.8\;\times \;\mathrm{Sex}\right)-5.4 \end{array}$$

Male = 1, Female = 0

For two of the PEs (Heitmann and Deurenberg equations), FFM was calculated by subtracting fat mass from total body mass. In defining the FFM and LBM, the authors in that study proposed that FFM and LBM could be used interchangeably. Mitchell et al. reported that FFM as estimated by Deurenberg equation had the smallest mean difference and overestimated FFM_DXA_ for overweight men but underestimated FFM_DXA_ for all other body mass index (BMI) subgroups [14]. The Heitmann and Janmahasatian equations, on the other hand, overestimated FFM_DXA_ across various BMI categories [14].

The addition of biochemistry variables might improve the performance of prediction equations but few studies have examined this. Creatine Kinase (CK) is found predominantly in skeletal muscle and serum levels were associated with the lean muscle mass [17]. There has only been one study evaluating the relationship between LBM and plasma creatine kinase activity (CK) and a weak and partial correlation (r < 0.262) between log CK and LBM was reported [18]. Serum albumin has also been reported to reflect protein reserve and lower albumin levels have been shown to be associated with loss of lean mass [19].

Therefore, the aims of this study were to develop and validate PEs for LBM with anthropometric and biochemistry variables against DXA.

## Methods

The Central Northern Adelaide Health Service Ethics of Human Research Committee approved this study. All participants provided written informed consent.

### Study cohorts

Four study cohorts were investigated in this study: a) the Cytokine, Adiposity, Sarcopenia and Ageing (CASA) cohort; b) the validation cohort (VC); c) the North West Adelaide Health Study (NWAHS) cohort and d) the Florey Adelaide Male Ageing Study (FAMAS) cohort. CASA was used to derive the PEs for LBM which included anthropometric and biochemistry variables. The selected LBM PEs were then validated in a second independent cohort, the VC (n = 52). As sarcopenia is more prevalent in older populations, validation of the best performing PE and other published FFM PEs (Heitmann, Janmahasatian and Deurenberg equations) were then undertaken in the larger population representative NWAHS and FAMAS cohorts (n = 2287, age ≥ 50 years).

#### CASA

195 population representative healthy subjects (age 18 to 83 years) were recruited from the western suburbs of Adelaide [20]. The inclusion criteria were: being aged 18 and above, able to comply with study protocol and weight stable over the last 3 months. We excluded those with a serious medical illness, an acute illness in the pass 3 months or in the 2 weeks following blood sampling, an inability to stop medications for 3 days prior to blood sampling, being in receipt of vaccinations and pregnancy. In undertaking the analysis, data from 7 subjects were excluded due to haemolysed or insufficient blood samples.

#### VC

This was a convenience sample of 52 healthy subjects (age 22 – 83 years) recruited through advertisement for another study [21]. Subjects with known medical illness including gastrointestinal disease or symptoms, significant respiratory, renal or cardiac disease and who were pregnant were excluded from this study.

#### NWAHS

This is a longitudinal study of community dwelling adults aged eighteen years and older. The population which is a representative biomedical cohort of predominantly of mixed European descent has been described in detail previously [22]. DXA scans were offered to NWAHS participants who were aged ≥ 50 years at follow up (median time = 4 years). Participants with complete anthropometric and DXA measurements at follow up (2004–06) aged ≥50 were included in this analysis (n = 1575).

#### FAMAS

This male only cohort has also been described in detail elsewhere [23]. The recruitment process was very similar to that used for the NWAHS and so the men in FAMAS were comparable with men in the same age groups from the NWAHS study and of mixed European descent [24]. DXA measurements at baseline (2002–2005) were obtained on 700 participants aged 50 years and over.

### Measurements

#### Anthropometry

Height (m) was measured without shoes using a wall-mounted SECA stadiometer to the nearest 0.1 cm. Weight (kg) was measured wearing light clothing to the nearest 0.1 kg (A&D FV platform scales 0.5 – 150 kg). Body mass index (BMI, weight/height^2^) was calculated. The healthy BMI for older people is said to be between 22–27 kg/m^2^[25]. Caucasians with BMI > 30 kg/m^2^ were classified as obese [26].

#### Dual Energy X-ray Absorptiometry (DXA)

DXA analysis in all cohorts measured 3 compartments of the total body composition; fat mass, LBM and bone mineral content. For the purpose of this study, LBM refers to soft tissues and muscle mass, but excludes fat and bone mass. CASA: A Lunar PRODIGY whole-body scanner (GE Medical Systems, Madison, WI), in conjunction with Encore 2002 software, was used to estimate LBM. The majority of subjects underwent DXA within 2 hours of attending the morning clinic when blood sampling occurred. VC: A Norland densitometer XR36 (Norland Medical Systems, Fort Atkinson, Wisconsin, USA), in conjunction with Illuminatus 4.2.4a software, was used to estimate LBM. The DXA was performed on a separate study day but within 2 weeks of blood sampling and given that the subjects were healthy, it is unlikely that there would have been significant change in body composition within that time frame. To account for differences between machines, LBM data from the VC had a correction factor applied to convert the data to Lunar equivalent [27]. NWAHS and FAMAS: The fan-beam Lunar PRODIGY (GE Medical Systems, Madison, WI) in conjunction with Encore 2002 software and a pencil-beam DPX + (GE Medical Systems, Madison, WI) in conjunction with LUNAR software version 4.7e were used. Cross-calibration analysis had been undertaken and no differences between these 2 densitometers were reported [28].

#### Blood analyses

For both the CASA and VC cohorts, a venous sample was obtained from each participant after an overnight fast. Both cohorts were asked to refrain from smoking, consuming alcohol or vigorous exercise in the 24 hours before the clinic appointment. Last regular medications were taken the day before and the morning dose was held until after venous sampling. For CASA, the blood was placed in ethylenediaminetetraacetic acid (EDTA) tubes and transported immediately to the Institute for Medical and Veterinary Sciences Laboratories (IMVS) in South Australia for analysis. The blood was centrifuged at 5000 rpm for 7 minutes and analyzed immediately at 37°C. For the VC, samples that had been centrifuged and stored at -70°C were transferred to be processed by the IMVS using the same methodology. The measured coefficients of variation (CV) were: alanine transferase (ALT, 1.98%), aspartate transaminase (AST, 2.8%),albumin (2.8%), creatinine (3%), lactate dehydrogenase (LDH, 2.2%), creatinine kinase (CK, 2.2%) and high sensitivity C-reactive protein (hsCRP, 1.4%). A Beckman Coulter AU 2700 was used to perform the blood analysis and the methods, reagents and calibration were as per manufacturer instructions.

#### Statistical analysis

Demographic characteristics in both groups were expressed as mean ± standard deviation (SD). Independent samples *t* test was used to compare means between the two cohorts. Differences between methods of LBM measurements in the same cohort were examined by paired *t* test. PEs for LBM were developed from CASA where the independent variable was DXA derived LBM. The initial 10 independent variables were gender, age, weight, height, body mass index, albumin, AST, LDH, CK and hsCRP. The best PEs (as assessed by adjusted R^2^: the proportion of the variance of the dependent variable accounted for by the independent variables, and adjusted for the number of independent variables) were developed considering up to 6 equations with *n* predictors. For each *n*, the PE for validation was selected by considering the adjusted R^2^ value and likely clinical utility. In the VC, LBM was calculated from the developed prediction equations (LBM_PE_) and compared with DXA derived LBM (LBM_DXA_).

The anthropometric PE was also compared to other known PEs [12,15,16] in the NWAHS and FAMAS cohorts.

To assess the accuracy and predictive performance of the prediction equations against LBM_DXA_, a regression analysis as proposed by Lin [29] was undertaken and the concordance correlation coefficient (*ρ*_c_) was derived. *ρ*_c_ measures how much the data deviates from the line of identity representing congruence between the methods. It is a product of Pearson correlation (*ρ*) and bias correction factor (*C*_*b*_): *ρ*_c_ = *ρ C*_b_[30].

In addition, to assess the level of agreement between the two methods, Bland-Altman analysis was performed to obtain the 95% limits of agreement [31]. Furthermore, the goodness of fit with root mean square error (RMSE) and bias (mean error [ME]) was also determined. RMSE and ME were calculated according to the method of Sheiner and Beal [32]. When the 95% confidence interval of the ME includes 0 (i.e. no error), it indicates that the model is not biased. In this study, mean difference was taken to be the same as ME. This gives an estimation of R^2^ and the standard error of the estimate [SEE]. SPSS 11.5 for Windows software (SPSS, Inc., Chicago, IL) and the R statistical language (R Foundation for Statistical Computing, Vienna, Austria) were used for the analyses. *P* < 0.05 was considered statistically significant.

## Results

The CASA and VC cohorts were similar in age (CASA mean [SD] 49.2 [17.0] vs. VC 50.6 [15.7] years), but younger than the NWAHS (64.7 [9.84] years) and FAMAS (62.3 [8.2] years) cohorts. The BMI (23.7 [2.3] vs. 26.7 [5.2] kg/m^2^) and CK (93.3 [54.7] vs. 114.3 [66.0] U/L), were significantly lower in the VC compared to the CASA. LDH (194.4 [37.8] vs. 175.0 [37.4] U/L) and albumin (40.4 [2.5] vs. 39.1 [3.1] g/L) were significantly higher in the VC compared to the CASA. No significant differences between the two cohorts were noted for hsCRP or LBM. The BMI of subjects in the NWAHS and FAMAS studies were higher at 28.2 [4.8] and 28.6 [4.6] kg/m^2^ respectively.

Based on adjusted R^2^ and potential clinical utility, the following PEs were selected for further validation in the VC:

$$\begin{array}{l} {\mathrm{LBM}}_{\mathrm{PE}1}=22.93+0.68\left(\mathrm{weight}\right)-1.14\left(\mathrm{BMI}\right)-0.01\left(\mathrm{age}\right)+9.94\left(\mathrm{if}\;\mathrm{male}\right)\;\mathrm{SEE}=3.61,R^{2}=90.7\\ {\mathrm{LBM}}_{\mathrm{PE}2}=22.06+0.67\left(\mathrm{weight}\right)-1.11\left(\mathrm{BMI}\right)+9.76\left(\mathrm{if}\;\mathrm{male}\right)+0.01\left(\mathrm{CK}\right)\;\mathrm{SEE}=3.56,R^{2}=91.0\\ {\mathrm{LBM}}_{\mathrm{PE}3}=21.19+0.67\left(\mathrm{weight}\right)-1.04\left(\mathrm{BMI}\right)+9.51\left(\mathrm{if}\;\mathrm{male}\right)-0.56\left(\mathrm{CRP}\right)+0.01\left(\mathrm{CK}\right)\;\mathrm{SEE}=3.47,R^{2}=91.4\\ {\mathrm{LBM}}_{\mathrm{PE}4}=23.17+0.64\left(\mathrm{weight}\right)-0.91\left(\mathrm{BMI}\right)+9.45\left(\mathrm{if}\;\mathrm{male}\right)+0.02\left(\mathrm{CK}\right)-0.58\left(\mathrm{CRP}\right)-0.02\left(\mathrm{LDH}\right)\;\mathrm{SEE}=3.38,R^{2}=91.9 \end{array}$$

Table 1 compares LBM_PE1-4_ to LBM_DXA_ in the VC. LBM predicted by all PEs was highly correlated with LBM_DXA_. Concordance correlations, a measure of the degree to which the data lie on the line of identity, were all around 0.9 and similar to the Pearsons correlation coefficient. All PEs over-estimated LBM_DXA_, ranging from 1.9% for PE_1_to 4.1% for PE_4_. The limits of agreement were similar for all PEs, approximately ± 15%. With increasing number of variables, there were reducing RMSE and mean error indicating improving precision and reducing bias. Because of the costs involved with blood investigations and the marginal benefits, only the anthropometric PE_1_was selected for further comparison in the combined NWAHS and FAMAS cohorts (Tables 2, 3 and 4). Furthermore, biochemistry was not readily available from those cohorts.

**Table 1:** Validation of PE LBM in healthy adults from the Cytokine, Adiposity, Sarcopenia and Ageing (CASA) study cohort (n = 195) against DXA derived LBM in the validation cohort (n = 52)

	**Mean (SD), kg**	**Mean error (95%CI), kg**	**P-value for mean error**	**R**	***ρ***_**c**_**(95% CI) [*C***_**b**_**]**	**95% limits of agreement**	**RMSE (95% CI), kg**
**Total (n = 52)**							
**LBM**_**DXA**_	46.2 (9.49)						
**LBM**_**PE1**_	48.1 (8.93)	1.88 ( 0.79, 2.97)	0.001	0.911*	0.891 (0.820, 0.935) [0.977]	-9.72, 5.96 (-20.7 to 12.6%)	4.32 (2.84, 5.80)
**LBM**_**PE2**_	47.9 (8.95)	1.69 (0.62, 2.75)	0.003	0.915*	0.899 (0.832, 0.940) [0.982]	-9.20, 5.83 (-19.9 to 12.6%)	4.15 (2.70, 5.60)
**LBM**_**PE3**_	47.7 (9.13)	1.50 (0.44 ,2.57)	0.006	0.917*	0.904 (0.840, 0.943) [0.986]	-8.99, 5.98 (-19.5 to 13.0%)	4.07 (2.63, 5.51)
**LBM**_**PE4**_	47.1 (8.96)	0.86 (-0.22, 1.94)	0.114	0.914*	0.908 (0.846, 0.946) [0.994]	-8.44, 6.72 (-18.3 to 14.6%)	3.93 (2.51, 5.35)

*P-value <0.001, R = correlation, SD = Standard Deviation.

RMSE = root mean squared prediction error, CI = confidence interval, R = Pearson Correlation Coefficient, C_b_ = Bias Correction Factor, ρ_c_ = Concordance Correlation Coefficient.

**Table 2:** **Performance of the CASA (**LBM_PE1_) **and previously published FFM prediction equations in the NWAHS and FAMAS cohorts (age 50 years and over) in the combined cohort and by gender**

	**Mean (SD), kg**	**Mean error (95%CI), kg**	**P-value for mean error**	**R**	***ρ***_**c**_**(95% CI) [*C***_**b**_**]**	**95% limits of agreement**	**RMSE (95% CI), kg**
**Total (n = 2287)**							
**LBM**_**DXA**_	50.62 (10.8)						
**Heitmann equation**	54.30 (10.7)	3.68 (3.53, 3.83)	<0.001	0.940*	0.888 (0.880, 0.896) [0.945]	-3.77, 11.1	5.24 (4.97, 5.51)
**Janmahasatian equation**	54.23 (11.0)	3.61 (3.46, 3.76)	<0.001	0.943*	0.884 (0.884, 0.899) [0.946]	-3.78, 11.0	5.17 (4.90, 5.44)
**Deurenberg equation**	50.64 (10.1)	0.02 (-0.14, 0.19)	0.777	0.931*	0.928 (0.923, 0.934) [0.998]	-7.89, 7.93	3.95 (3.70, 4.20)
**LBM**_**PE1**_	51.36 (10.6)	0.74 (0.59, 0.89)	<0.001	0.942*	0.939 (0.934, 0.944) [0.998]	-6.58, 8.06	3.73 (2.48, 4.98)
**Men (n = 1436)**							
**LBM**_**DXA**_	57.09 (7.50)						
**Heitmann equation**	60.56 (7.80)	3.46 (3.25. 3.67)	<0.001	0.863*	0.782 (0.764, 0.800) [0.906]	-11.5, 4.57	5.30 (4.93, 5.67)
**Janmahasatian equation**	61.18 (6.80)	4.09 (3.89, 4.29)	<0.001	0.852*	0.728 (0.707, 0.747) [0.853]	-12.0, 3.82	5.69 (5.32, 6.06)
**Deurenberg equation**	56.76 (6.80)	- 0.34 (-0.55, -0.12)	0.002	0.838*	0.834 (0.818, 0.848) [0.995]	-7.92, 8.60	4.14 (3.85, 4.43)
**LBM**_**PE1**_	58.22 (6.11)	1.12 (0.92, 1.33)	<0.001	0.851*	0.822 (0.806, 0.837) [0.851]	-6.78, 9.02	4.11 (3.80, 4.42)
**Women (n = 851)**							
**LBM**_**DXA**_	39.70 (5.30)						
**Heitmann equation**	43.74 (5.55)	4.04 (3.83, 4.26)	<0.001	0.833*	0.651 (0.620, 0.680) [0.782]	-10.3, 2.26	5.12 (4.75, 5.49)
**Janmahasatian equation**	42.50 (5.39)	2.81 (2.60, 3.01)	<0.001	0.837*	0.722 (0.693, 0.749) [0.872]	-8.91, 3.29	4.14 (3.83, 4.45)
**Deurenberg equation**	40.32 (4.90)	0.63 (0.39, 0.87)	<0.001	0.759*	0.751 (0.721, 0.779) [0.990]	-7.75, 6.49	3.61 (3.29, 3.93)
**LBM**_**PE1**_	39.78 (5.11)	0.08 (-0.12, 0.28)	0.433	0.835*	0.835 (0.813, 0.854) [0.999]	-5.91, 6.07	2.99 (2.74, 3.24)

Mean Error = DXA-PE; LBM, Lean Body Mass; DXA, Dual X-ray absorptiometry; RMSE, Root Mean Square Error; CI, Confidence interval; SD, Standard Deviation; R, Pearson Correlation; C_b_ = Bias Correction Factor; ρ_c_ = Concordance Correlation Coefficient; *p-value <0.001.

**Table 3:** **Performance of the CASA (**LBM_PE1_) **and previously published FFM prediction equations in the NWAHS and FAMAS cohorts (age 50 years and over) across various age groupings**

	**Mean (SD), kg**	**Mean error (95%CI), kg**	**P-value for mean error**	**R**	***ρ***_**c**_**(95% CI) [*C***_**b**_**]**	**95% limits of agreement**	**RMSE (95% CI), kg**
**Age 50–64, years (n = 1265)**							
**LBM**_**DXA**_	52.27 (11.2)						
**Heitmann equation**	56.47 (10.8)	4.20 (3.99, 4.40)	<0.001	0.944*	0.879 (0.868, 0.890) [0.932]	-11.6, 3.23	5.60 (5.26, 5.95)
**Janmahasatian equation**	55.62 (11.2)	3.35 (3.15, 3.55)	<0.001	0.948*	0.907 (0.897, 0.915) [0.956]	-10.6, 3.85	4.92 (4.61, 5.23)
**Deurenberg equation**	53.15 (10.0)	0.87 (0.66, 1.09)	<0.001	0.938*	0.929 (0.921, 0.936) [0.990]	-8.72, 6.98	4.02 (3.73, 4.31)
**LBM**_**PE1**_	52.77 (10.7)	0.50 (0.30, 0.70)	<0.001	0.948*	0.946 (0.939, 0.951) [0.998]	-6.68, 7.68	3.62 (3.36, 3.88)
**Age 65–79, years (n = 882)**							
**LBM**_**DXA**_	49.09 (9.91)						
**Heitmann equation**	52.23 (10.0)	3.14 (2.90, 3.38)	<0.001	0.933*	0.887 (0.873, 0.899) [0.951]	-10.5, 4.18	4.82 (4.35, 5.29)
**Janmahasatian equation**	53.03 (10.5)	3.93 (3.69, 4.18)	<0.001	0.933*	0.862 (0.846, 0.876) [0.925]	-11.5, 3.66	5.46 (4.97, 5.95)
**Deurenberg equation**	48.19 (9.14)	-0.90 (-1.15, -0.65)	<0.001	0.924*	0.916 (0.905, 0.926) [0.993]	-6.70, 8.50	3.90 (3.45, 4.35)
**LBM**_**PE1**_	50.20 (10.2)	0.98 (0.73, 1.22)	<0.001	0.929*	0.925 (0.915, 0.934) [0.995]	-6.57, 8.53	3.90 (3.48, 4.32)
**Age ≥80, years (n = 140)**							
**LBM**_**DXA**_	44.48 (8.64)						
**Heitmann equation**	46.71 (9.20)	2.23 (1.60, 2.85)	<0.001	0.929*	0.902 (0.868, 0.928) [0.969]	-9.05, 4.59	4.06 (3.20, 4.92)
**Janmahasatian equation**	48.46 (10.1)	3.97 (3.29, 4.66)	<0.001	0.936*	0.850 (0.806, 0.883) [0.906]	-11.4, 3.46	5.43 (4.31, 6.55)
**Deurenberg equation**	42.46 (8.41)	-2.03 (-2.58, -1.48)	<0.001	0.937*	0.911 (0.880, 0.934) [0.971]	-3.97, 8.03	3.61 (2.85, 4.37)
**LBM**_**PE1**_	45.84 (9.81)	1.36 (0.80, 1.93)	<0.001	0.941*	0.923 (0.897, 0.943) [0.981]	-5.39, 8.11	3.63 (2.90, 4.36)

Mean Error = DXA-PE; LBM, Lean Body Mass; DXA, Dual X-ray absorptiometry; RMSE, Root Mean Square Error; CI, Confidence interval; SD, Standard Deviation; R, Pearson Correlation; C_b_ = Bias Correction Factor; ρ_c_ = Concordance Correlation Coefficient; *p-value <0.001.

**Table 4:** **Performance of the CASA (**LBM_PE1_) **and previously published FFM prediction equations in the NWAHS and FAMAS cohorts (age 50 years and over) across various body mass index groupings**

	**Mean (SD), kg**	**Mean error (95%CI), kg**	**P-value for mean error**	**R**	***ρ***_**c**_**(95% CI) [*C***_**b**_**]**	**95% limits of agreement**	**RMSE (95% CI), kg**
**BMI < 22 kg/m**^**2**^**(n = 135)**							
**LBM**_**DXA**_	42.45 (8.85)						
**Heitmann equation**	44.85 (7.65)	2.40 (1.85, 2.96)	<0.001	0.932*	0.885 (0.847, 0.914) [0.949]	-4.12, 8.92	4.04 (3.21, 4.87)
**Janmahasatian equation**	43.72 (9.26)	1.27 (0.77, 1.77)	<0.001	0.946*	0.937 (0.914, 0.955) [0.989]	-4.65, 7.19	3.21 (2.55, 3.87)
**Deurenberg equation**	41.26 (8.04)	-1.18 (-1.77, -0.60)	<0.001	0.921*	0.909 (0.876, 0.933) [0.986]	-8.04, 5.68	3.62 (2.86, 4.36)
**LBM**_**PE1**_	43.52 (9.04)	1.08 (0.57, 1.59)	<0.001	0.944*	0.937 (0.913, 0.955) [0.993]	-4.92, 7.08	3.18 (2.53, 3.83)
**BMI 22- < 27 kg/m**^**2**^**(n = 847)**							
**LBM**_**DXA**_	47.45 (9.18)						
**Heitmann equation**	50.67 (8.67)	3.22 (2.99, 3.44)	<0.001	0.933*	0.874 (0.860, 0.888) [0.938]	-3.42, 9.86	4.62 (4.26,4.98)
**Janmahasatian equation**	50.81 (9.71)	3.36 (3.13, 3.59)	<0.001	0.937*	0.880 (0.866, 0.893) [0.939]	-3.41, 10.1	4.77 (4.39, 5.15)
**Deurenberg equation**	47.91 (8.68)	0.45 (0.22, 0.68)	0.001	0.928*	0.925 (0.915, 0.934) [0.997]	-6.42, 7.32	3.46 (3.16, 3.76)
**LBM**_**PE1**_	48.64 (9.45)	1.19 (0.96, 1.41)	<0.001	0.938*	0.930 (0.920, 0.938) [0.992]	-5.41, 7.79	3.51 (3.20, 3.82)
**BMI 27- < 30 kg/m**^**2**^**(n = 596)**							
**LBM**_**DXA**_	52.00 (9.83)						
**Heitmann equation**	55.65 (9.48)	3.65 (3.36, 3.95)	<0.001	0.929*	0.867 (0.847, 0.883) [0.933]	-3.65, 10.9	5.16 (4.69, 5.63)
**Janmahasatian equation**	56.11 (9.75)	4.12 (3.83, 4.41)	<0.001	0.932*	0.857 (0.837, 0.874) [0.919]	-3.08, 11.3	5.47 (4.97, 5.97)
**Deurenberg equation**	52.58 (9.23)	0.59 (0.30, 0.88)	<0.001	0.928*	0.925 (0.912, 0.935) [0.996]	-6.72, 7.90	3.70 (3.35, 4.05)
**LBM**_**PE1**_	52.80 (9.69)	0.81 (0.52, 1.09)	<0.001	0.933*	0.929 (0.918, 0.939) [0.997]	-6.37, 7.99	3.67 (3.31, 4.03)
**BMI ≥30 kg/m**^**2**^**(n = 709)**							
**LBM**_**DXA**_	54.80 (11.7)						
**Heitmann equation**	59.30 (11.7)	4.50 (4.19, 4.80)	<0.001	0.937*	0.867 (0.847, 0.883) [0.933]	-3.80, 12.8	6.12 (5.53, 6.71)
**Janmahasatian equation**	58.93 (11.0)	4.13 (3.83, 4.43)	<0.001	0.937*	0.857 (0.837, 0.974) [0.919]	-4.02, 12.3	5.80 (5.22, 6.38)
**Deurenberg equation**	54.07 (10.6)	-0.74 (-1.08, -0.39)	<0.001	0.917*	0.925 (0.912, 0.935) [0.996]	-10.0, 8.55	4.70 (4.14, 5.26)
**LBM**_**PE1**_	54.88 (11.3)	0.08 (-0.23, 0.38)	0.628	0.936*	0.929 (0.918, 0.939) [0.997]	-8.15, 8.31	4.11 (3.61, 4.61)

Mean Error = DXA-PE; LBM, Lean Body Mass; DXA, Dual X-ray absorptiometry; RMSE, Root Mean Square Error; CI, Confidence interval; SD, Standard Deviation; R, Pearson Correlation; C_b_ = Bias Correction Factor; ρ_c_ = Concordance Correlation Coefficient.

*p-value <0.001.

Table 2 compares the performance of various PEs including PE_1_ against LBM_DXA_ in the total combined NWAHS and FAMAS cohorts as well as in the two gender groups, men and women. All PEs over-estimated the LBM_DXA_ in the total group. PE_1_ demonstrated a lower mean error and RMSE score than the Heitmann and Janmahasatian equations in the total population, men and women cohorts. The Deurenberg equation performed the best in the total population with the lowest mean error and RMSE. However, when reviewed within gender groups, PE_1_ performed better than the Deurenberg equation in women where both equations over-estimated LBM. In men, the Deurenberg equation under-estimated LBM whilst all other equations over-estimated LBM.

Table 3 compares the performance of the various PEs across age groups (60–64, 65–79, ≥80). PE_1_ consistently over-estimated LBM_DXA_ across the age groups but performed better (lowest ME, RMSE values and higher concordance correlation coefficient) than the Janmahasatian and Heitmann equations. The Deurenberg equation did not perform as well as PE_1_ in the 50- < 65 years age group and the ≥ 80 years age group and over-estimated LBM in the 50- < 65 years age group but under-estimated LBM in the other two age groups.

Table 4 compares the performance of the various PEs across various BMI groups. Once again, PE_1_ has the smallest ME and RMSE compared with the Janmahasatian and Heitmann equations across all the BMI groups analyzed but all of these consistently over-estimated LBM_DXA_ across the various BMI groups. PE_1_, in comparison with the Deurenberg equation has a lower ME and RMSE in the obese BMI (>30 kg/m^2^) and underweight BMI (< 22 kg/m^2^) groups. Interestingly, the Deurenberg equation has less bias and better precision than PE_1_ in predicting LBM_DXA_ in the 22-27 kg/m^2^ BMI group. The Deurenberg equation overestimated LBM_DXA_ except in the underweight and obese categories.

## Discussion

In this study, prediction equations for LBM were developed and validated. It was hypothesized that the addition of biochemistry variables would result in an improvement in the performance of the PEs and this was seen. However, the improvement was marginal and insufficient to justify the additional costs.

A significant finding from this study wasthe development of a new anthropometric PE (PE_1_) for LBM: *LBM = 22.932326 + 0.684668 (weight) -1.137156 (BMI) -0.009213 (age) + 9.940015 (if male)*. The close approximation to LBM_DXA_ generated by this equation was reflected by its small bias (ME = 0.74 kg) and precision (RMSE = 3.73 kg). It overestimated LBM_DXA_ across gender, age and BMI groups. This PE may be useful in care settings where access to DXA may be limited, providing clinicians a practical alternative to assess LBM. Furthermore, it also provides a bedside option in hospitals for ill and frail patients where transport for DXA assessment may be difficult. Whilst BIA may be simple technique to be used at the beside, BIA may be affected by clinical factors such as ascites, hydration status, food intake and exercise and cannot be used in older people with pacemakers [11]. Skin fold measurements may be a cheaper option but the accuracy is operator dependent and the loss of subcutaneous tissue in older people may also affect accuracy [33].

Interestingly, the Deurenberg equation appeared to have less bias with a ME of 0.02 kg but similar precision with a RMSE of 3.95 when compared to the newly developed PE. However, across gender, age and BMI groups, it at times over-estimated and at other times under-estimated the LBM_DXA_[14]. The newly developed PE_1_ appeared to have better precision (smaller RMSE) and less bias (lower ME) than the Deurenberg equation only in women and in obese older individuals. In clinical settings where the dose normalization to LBM is required, an overestimation of LBM could potentially lead to higher incidence of dose limiting toxicity. Sarcopenia was an important predictor of toxicity in women with metastatic cancer and colon cancer receiving chemotherapy [4,6]. It was suggested that chemotherapy dose normalization to LBM may reduce the excess toxicity in women. PE_1_ in our study potentially offers a more accurate estimation of LBM over Deurenberg equation in women and obese individuals and may have clinical utility in this two patient population groups.

This study had several limitations. Only 6% of the study population was under-weight with a BMI < 22 kg/m^2^ and therefore, it remains important to validate this newly developed PE in an under-weight population where sarcopenia is likely to be common. Furthermore, only Caucasians were studied and therefore generalizing these results to other ethnic communities is not possible and ethnic specific PEs will need to be developed. Different DXA machines were used in the CASA and VC cohort studies. This may have affected the results as even in the same person, reported measurements of the same tissue mass can be different with different DXA machines [34]. The researchers adjusted for the difference between the machines in the validation aspects of this study but clearly, it would have been preferable to use the same DXA machine in both cohorts. The use of other anthropometry measurements such as calf or arm circumference may improve the performance of prediction equations and needs to be explored in future studies.

## Conclusions

This study describes the development of a new prediction equation for LBM as estimated by DXA. This new PE consistently over-estimates across gender, age and BMI groups. There remains a need to confirm these findings in older and leaner cohorts, cohorts with diseases (e.g. renal failure), as well as other cohorts with varying ethnicity. The anthropometric PE is an alternative when access to DXA is difficult and this might occur with home bound frail older people as well as people residing in rural areas. The availability of simple and accuratemethods to estimate LBM might be the necessary catalyst required to support better prescribing to limit toxicity in the oncology setting.

## Competing interests

TV, JF, JCW, IC, RA and GW have no conflicts of interest; SY received scholarship from University of Adelaide, Faculty of Health Sciences, Divisional Scholarship to support his PhD studies; RV received research grants from: i) University of Adelaide, Faculty of Health Sciences, Establishment Grant; ii) University of Adelaide, Faculty of Health Sciences, Early Career Grant; iii) Vincent Fairfax Family Foundation Research Fellowship through the Royal Australasian College of Physicians; iv) Bernie Lewis Foundation Grant through the Hospital Research Foundation; RVis a member of the Nestle Nutrition Australia Malnutrition In The Elderly Board and receives a honorarium for this activity. She has previously also been a member of Mini Nutritional Assessment (MNA) International Group to refine the MNA and more recently attended the International Consensus Meeting On Protein Intake In The Elderly and both meetings were undertaken independently but supported by an educational grant from Nestle Pty Ltd. She has no other relationships or activities that could appear to have influenced the submitted work. LCW has provided consultancy services to ImpediMed Ltd. The authors declare that they have no competing interests.

## Authors’ contributions

RV, TV, RA and SY contributed to the initial study design. SY contributed to the data collection, data entry and statistical analysis. JF undertook the statistical analysis required to develop the PEs. LW performed the LBM data correction to adjust differences between dual x-ray absorptiometry machines in this study. All authors contributed to the interpretation of the analysis and preparation of this manuscript. All authors read and approved the final manuscript.

## Pre-publication history

The pre-publication history for this paper can be accessed here:

http://www.biomedcentral.com/2050-6511/14/53/prepub
